# Can microcredit help improve the health of poor women? Some findings from a cross-sectional study in Kerala, India

**DOI:** 10.1186/1475-9276-7-2

**Published:** 2008-01-10

**Authors:** KS Mohindra, Slim Haddad, D Narayana

**Affiliations:** 1 Department of Health Care and Epidemiology, University of British Columbia, Vancouver, Canada; 2 Centre de recherche du Centre Hospitalier de l'Université de Montréal, Canada; 3 Groupe de Recherche Interdisciplinaire en santé, Université de Montréal, Canada; 4 Centre for Development Studies, Thiruvananthapuram, India

## Abstract

**Background:**

This study examines associations between female participation in a microcredit program in India, known as self help groups (SHGs), and women's health in the south Indian state of Kerala. Because SHGs do not have a formal health program, this provides a unique opportunity to assess whether SHG participation influences women's health via the social determinants of health.

**Methods:**

This cross-sectional study used special survey data collected in 2003 from one Panchayat (territorial decentralized unit). Information was collected on women's characteristics, health determinants (exclusion to health care, exposure to health risks, decision-making agency), and health achievements (self assessed health, markers of mental health). The study sample included 928 non elderly poor women.

**Results:**

The primary finding is that compared to non-participants living in a household without a SHG member, the odds of facing exclusion is significantly lower among early joiners, women who were members for more than 2 years (OR = 0.58, CI = 0.41–0.80), late joiners, members for 2 years and less (OR = 0.60, CI = 0.39–0.94), and non-participants who live in a household with a SHG member (OR = 0.53, CI = 0.32–0.90). We also found that after controlling for key women's characteristics, early joiners of a SHG are less likely to report emotional stress and poor life satisfaction compared to non-members (OR = 0.52, CI = 0.30–0.93; OR = 0.32, CI = 0.14–0.71). No associations were found between SHG participation and self assessed health or exposure to health risks. The relationship between SHG participation and decision-making agency is unclear.

**Conclusion:**

Microcredit is not a panacea, but could help to improve the health of poor women by addressing certain issues relevant to the context. In Kerala, SHG participation can help protect poor women against exclusion to health care and possibly aid in promoting their mental health.

## Background

The deep connections between poverty and health continue to be the source of intensive investigations in the twenty-first century, especially in low and middle-income countries where the burden of illness is the heaviest [1,2]. Reducing social inequalities in health in general and the burden of ill health among the poor in particular are currently driving many global health research and activist agendas. In addition to ensuring that the poor have access to essential health services, there is a need for complementary interventions in poverty alleviation that have positive effects on health [1].

This paper aims to explore the associations between health and female participation in a self help group (SHG), a microcredit scheme in India. Microcredit aims to extend access to credit to the poor, especially poor women, in order to generate income for participants and their families [3]. These groups were designed as a poverty alleviation strategy and as a means to increase women's access to resources and decision-making powers. And researchers have begun to explore whether microcredit participation may also benefit the health of poor women [4-6]. The bulk of studies focus on a few popular schemes in Bangladesh, notably BRAC and the Grameen Bank. As programs are rooted in the context in which they are being implemented, the available evidence is limited in its generalizability. To gain a broader understanding, there is a need to explore other types of microcredit programs, which vary in their typology [7], across contexts with different epidemiological and socioeconomic profiles. This paper aims to contribute to this body of evidence in the south Indian state of Kerala.

Kerala is widely known for its health achievements despite modest economic growth [8]. Women measure up well in basic capabilities: fertility rates are below replacement levels, life expectancy is 76 years, and literacy rates are over 90% [9]. These achievements are generally attributed to progressive public policies, social reforms by pre-independence rulers, and the earlier influence of matrilineal communities that led to greater freedom among women [10]. Less well known are the challenges faced by women. With an aging population, chronic illness and disability play more prominent roles in women's lives, yet women's non-fatal health status is not well documented [11]. Rising health care costs and a lack of social protection in Kerala are leading to financial burdens, especially among the poor [12], who are vulnerable to exclusion from health care, indebtedness and impoverishment. Threats specific to women's physical and mental health have also been noted, such as the spread of dowry and dowry-related crimes, domestic violence, and male alcohol abuse [13,14]. Meeting the health needs of women – including their mental health – remains an important challenge in this state.

SHGs were launched in India by the National Bank for Agriculture and Rural Development (NABARD), with the support of non governmental organizations (NGOs); the predicted coverage is at least one third of the rural population by 2008 [15]. SHGs were promoted as an alternative to previous supply-led, top-down poverty alleviation strategies. SHGs adopt the position that the poor are agents and that group members themselves should decide loan criteria and identify their own projects and activities. SHGs are linked to commercial banks and group solidarity is used as collateral, enabling access to resources much larger than the group's savings. In Kerala, a SHG program supported by local government, known as *kudumbrasree*, is also underway. These programs emphasize the empowerment of women [7] and engage in a range of activities, including income-generation, skills training, and women's rights and awareness campaigns. The weekly meetings attended by women also provide the opportunity for social support and sharing of knowledge and skills. But SHGs are not affiliated with any formal health program or service. This provides a unique opportunity to first, assess whether SHG participation – in the absence of formal health programs – influences women's health via the social determinants of health, and second, to explore potential avenues in which SHGs may extend their activities to meet health needs of women in the community.

In this paper, we test two hypotheses derived from a theoretical framework developed elsewhere [16], relevant to the Kerala context. First, we explore whether among poor women, SHG participants will have greater opportunities for health compared to non-participants. We focussed on three opportunities for health that are likely to be positively influenced by SHG participation: exclusion to health care, exposure to health risks, and decision-making agency. Second, among poor women, SHG members will have better health achievements (self assessed health, markers of mental health) compared to non-participants, mediated through access to health care, exposure to health risks, and decision-making agency.

## Methods

### Setting and SHG program

Our study was conducted in Kerala's district of Wayanad. The population is largely dependent on agriculture with the main crops being coffee and paddy. The study site was a single Panchayat (territorial decentralised unit) with a land area of 31.75 sq km and a population of 16,110 individuals. Forty-three percent of households were classified as below the poverty line at the time of the study in 2003. The classification of households into poor and non poor in India is not based on a direct assessment of income. Households are classified below the poverty line according to a series of indicators, including education, presence of disability, social group affiliation, dwelling type, land and livestock owned, formal training for skill development of household members, and consumption expenditure.

Women's health is unevenly distributed in the Panchayat; the prevalence of poor health was higher among women of low socioeconomic position and with low caste affiliations [11]. There is a high density of public and private health care facilities and geographical access to care is evenly distributed among the population. The poor, however, often face difficulties in obtaining services because of financial barriers. Rapidly rising health care costs have been observed in Kerala [17]. And although the services are supposedly free of charge, deficiencies in the quality of the public system force the poor to turn to the private sector to purchase medication and other services.

In 1995, SHGs began operating in the area. This initiative emerged from within the community, initially led by a small group of women with local NGO support. Following this, there was an incremental expansion of SHGs. Later, in 2002, *kudumbrasree *was introduced. Both networks follow similar procedures: small groups of women engage in savings and loan activities; their weekly contributions are deposited in a commercial bank. After an initial savings period (typically 6 months), members are eligible to take loans. Each SHG sets their interest rates and procedures for loan allocation. Any woman 18 years and older can participate, whether she is from a household that is below or above the poverty line. Due to the similarities of the NGO supported groups and *kudumbrasree*, we do not discriminate between the networks in this study (hereafter both networks are collectively referred to as SHGs). Basic information on participation and loans are presented in Table 1.

**Table 1:** Basic data on SHGs: participants, savings and loan activities in Kottathara Panchayat, Kerala, 2003

Number of adult women living in Kottathara^a^	5584
Number (and %) of adult women participating in SHG	2034 (36%)
Mean contributions to savings per year in Indian Rupees (approximate equivalent in US dollars)	650.00 (15.00)
Median contributions to savings per year in Indian Rupees (approximate equivalent in US dollars)	520.00 (12.00)
% of members who received at least one loan^cd^	73%
Mean number of loans taken^d^	1.91
Median number of loans taken^d^	1.00
Mean amount of loans taken in Indian Rupees (approximate equivalent in US dollars)^d^	3345.00 (78.00)
Median amount of loans taken in Indian Rupees (approximate equivalent in US dollars) ^d^	2000.00 (46.00)

Data source : authors.

^a^Adult women here are considered 18 years and older; 18 is the age that women can join SHGs and is also the legal age for marriage. Females under 18 can participate in 'child' SHGs.

^b ^includes women who participate in both kudumbrasree and NGO-supported groups.

^c^This number includes only those women who are eligible for a loan, which is after 6 months of participation.

^d^The recall period is over the duration of women's participation.

### Sources of data and study population

Cross-sectional data were used from a household survey implemented in 2003 as part of a our (Centre for Development Studies and Université de Montréal) action research project. The project obtained ethical approval by the Université de Montréal Ethics Committee on April 25, 2003. Trained local female surveyors canvassed all 3,352 households identified in the Panchayat. The household questionnaire has several modules, including questions pertaining to demographics, socioeconomic characteristics, health, and SHG participation. One woman from each household, the head or spouse of the head, was also invited to participate in a women's well-being module, which collected information on markers of mental health and women's decision-making agency. To maintain privacy, women were encouraged to respond to this module separately from other household members.

The study sample included non-elderly females (18 to 59 years) who responded to the women's well-being module for themselves. Paniya women (N = 647), a particularly deprived and socially marginalized group, were not included in the study. Large socioeconomic and cultural differences between groups can increase difficulties in comparing health status when using self reported health [18]. We detected that Paniyas were rating their health in a distinct way that underestimated their health; therefore, we did not include Paniya women in our analysis [11]. Finally, because our hypotheses relate specifically to poor women, we only included women from households below the poverty line.

### Measurements

#### Health determinants and health achievements

Exclusion to health care was measured at the household level. This is only a proxy of women's exclusion to care as gender-based barriers may affect women's access to and utilization of health care. However, gender discrimination of this form is less prevalent in Kerala compared to other regions in India. To measure exclusion to care, we used 8 questions that explored 8 situations in which household members may have been excluded from care during the previous twelve months (see Table 2). Exclusion was defined as at least one situation of exclusion over the past 12 months (35% of women's households). Decision-making agency was measured by whether a woman's husband (or male relative) was the sole decision-maker in five key areas (see Table 2). Both female only and joint decision-making (a woman and her husband) were considered to reflect a high level of decision-making agency. Male decision-making was defined as whether a woman reported at least one situation in which her husband or male relative was the sole decision-maker (12% of women). Exposure to health risks was assessed through self reports of exposure to risks in 2 situations (See Table 2). Exposure to health risks was measured by a woman reporting at least one situation in which she was exposed to a risk (22% of women).

**Table 2:** Health determinants used in the study, items and decision criteria for variable

Health determinant	Items	Decision criteria for variable
Exclusion to health care	1. A child in the family was sick but was unable to obtain the required health care. 2. An adult in the family was sick, but was unable to obtain the required health care. 3. An elderly member in the family was sick but was unable to obtain the required health care 4. A family member, having a chronic illness, had to stop his/her treatment for a certain period of time. 5. A doctor recommended a hospitalisation for a family member but we did not have it done. 6. A doctor recommended a surgery for a family member but we did not have it done. 7. A doctor recommended a hospitalisation for a family member but we postponed it. 8. A doctor recommended a surgery for a family member but we postponed it.	At least one situation of exclusion versus no exclusion.

Exposure to health risks	1. Exposed at work to any particular health risk. 2. Exposed in the home to any particular health risk.	At least one situation of exposure to a health risk versus no exposure.

Decision-making agency	1. Seeking health care of family member 2. Daily household expenditures 3. Child's education in school 4. Family planning 5. Voting in an election	At least one situation of male decision-making versus no male-decision making.

We used four measures of health achievements: two measures of self assessed health and two markers of mental health. Self assessed health was measured first by asking respondents to rate their overall perceived health based on a five point likert scale, which was converted into a binary variable (very bad and bad health versus good, very good, and excellent health). Second, limitations in activities of daily living (ADLs) were measured by asking respondents to rate their level of limitations for two different sets of activities, physically demanding activities and moderately demanding activities. A single indicator was computed by summing the responses for the two sets of activities and it was converted into a binary variable (limitations versus no limitations). This variable does not capture the full range and variation of activities that a woman may need to perform to lead a healthy life, but does provide a rough indication of the functional status of the woman. Markers of mental health were based on questions adopted from a survey in South Asia [19]. First, women were asked to report the frequency in which they experienced disturbances in mental peace (almost daily, occasionally, rarely, never), a response of almost daily or occasional disturbances was used as an indication of emotional stress. Second, women were asked about their life satisfaction (unsatisfied, moderately satisfied, greatly satisfied), the variable was converted into a binary variable of unsatisfied versus moderately or greatly satisfied. These variables were pre-tested and used in a study in Bangladesh [19], although the authors do not include any information on the validity and reliability of these questions.

### SHG participation

SHG participation was measured as a three level variable in order to consider duration of participation – the longer a woman has participated, the more likely she may benefit [16]. The categories were: early joiners (members for more than 2 years), late joiners (members for 2 years and less), and non-members. In our models for exclusion to health care, we further distinguished non-members between women with and without another household member participating in a SHG because exclusion was measured at the household level; therefore, women who have another household member participating in a SHG could indirectly benefit. We gathered additional background information on SHG participation, including the number and purpose of loans of each woman and the reasons for non-participation at the household level.

### Women's characteristics

A variety of indicators can be used to measure socioeconomic position. We used three indicators that we believe are pertinent for Indian agrarian societies: education, employment status, and size of land holdings [11]. We measured the size of household landholdings in cents (100 cents equals one acre). We also included the specific caste of the household, which was surveyed and categorized using the conventional three-way classification system adopted in Kerala, which ranks Hindu castes and other religions. The first category, at the bottom of the caste hierarchy, includes both Scheduled Castes (SC) and Scheduled Tribes (ST), or "SC/ST". Next is a residual category of lower castes and Muslims, known as other backward castes or "OBC". The highest ranking group are the upper or "forward castes", including Christians. Finally, we controlled for age and the position of women in the household (i.e. head or spouse of the head of household).

### Data analysis

**Figure 1:**
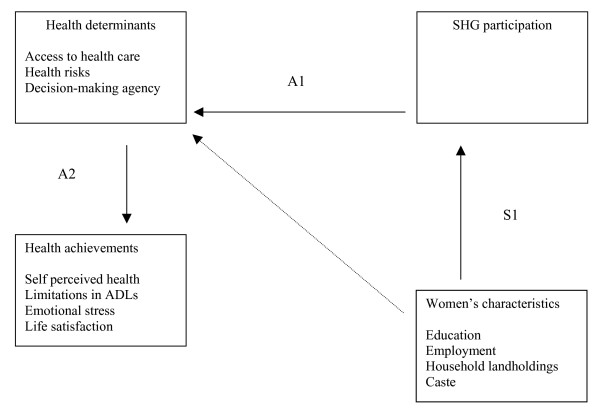
Determinants of SHG participation (S1) and associations (A1, A2) between SHG participation, women's characteristics, health determinants, and health achievements, and variables used in the study.

Data analysis followed a three step process (Figure 1). To illuminate potential selection bias in our sample we began by examining the socioeconomic and demographic characteristics of SHG participation (S1). Basic cross-tabs with chi-squares were used to develop a portrait of the characteristics of participation, followed by multinomial logistic regression analyses to test whether SHG participation (early joiner, late joiner, non-participant) varied according to these characteristics. We also assessed reasons reported for non-participation and the number of and purposes for loans among SHG members.

We then tested the study's hypotheses: SHG participants face less exclusion to health care, lower health risks, and are less likely to engage in male dominated decision-making (A1); SHG participants have greater health achievements, mediated by the determinants of health examined in A1 (A2). Due to the nature of the variables (categorical or scores with little distribution), a series of binomial logistic regressions were performed for each of the dependent variables. Associations between participation and health determinants (A1) adopted a three step approach to modelling. In step one, only SHG participation was included (Model 0), allowing us to assess the independent effects of SHG participation. Steps two and three adopted a sequential approach: socioeconomic characteristics and caste were entered in the model (Model 1), then, SHG participation was added (Model 2). We examined the sequential models in two ways. First, we employed goodness-of-fit tests to observe whether SHG participation significantly improved Model 1 by testing whether the deviance was statistically significant between Model 1 and Model 2. Second, we examined the odds ratios to assess the associations between being an early or late joiner and each of the women's characteristics and the dependent variable.

Modelling of health achievements (A2) followed a similar approach using four steps. In step one, only SHG participation was entered (Model 0). A sequential approach was then followed: socioeconomic characteristics and caste were entered in the model (Model 1), SHG participation was added (Model 2), and finally the determinants of health (exclusion, decision-making, health risks) were added as explanatory variables (Model 3). This approach allowed us to test the effect of our variable of interest, SHG participation, on our dependent variables, while assessing the influence of other determinants of health. Deviances comparing Model 1 and Model 2 indicate whether or not SHG participation significantly contributes to the model and deviances comparing Model 2 and Model 3 indicate whether, globally, the block of determinants of health significantly improve the model. Odds ratios were examined as in A1. All multivariate analyses controlled for age and women's position in the household. Data analysis was performed using SPSS version 14.0 [20].

## Results

From the 4,196 non-elderly adult, non-Paniya women identified in the Panchayat, we included 2,364 women who responded to the women's well-being module. We then limited the population to women from households below the poverty line, yielding a sample size of 928 women.

### Non-participation

There are 336 women who did not participate in a SHG, of which 100 women are from households already containing a SHG participant. The main reason reported for non-participation was financial barriers (over 50%), confirming the need to control for socioeconomic characteristics in our analyses. Three percent of households reported ill-health as a reason for non-participation. Sensitivity analysis showed that including women from these households did not affect our results; therefore, these women were retained in the analysis.

### SHG participation and loans

Over half of the women are members of a SHG (150 early joiners and 442 late joiners). Almost 75% of members had received at least one loan (91% of early joiners and 67% of late joiners). In addition to productive activities, 42% of women who had ever received a loan reported health consumption purposes.

### SHG participation and socioeconomic and demographic characteristics (S1)

Women are less likely to be a SHG member if they are in the youngest or oldest age category and had less than a high school education (Table 3). There are no significant associations between SHG participation and employment status, household landholdings, or caste.

**Table 3:** Demographic and socioeconomic characteristics of women, by SHG participation (percentages)

Characteristic		Not member N = 336	Member N = 592	
			
			Late joiner (< = 2 years) N = 442	Early joiner (> 2 years) N = 150
Age of woman (years)***	30 and under (n = 227)	38.3	48.5	13.2
	31–44 (n = 418)	28.0	52.4	19.6
	45–59 (n = 283)	46.6	39.9	13.4
Relationship to head	Head (n = 126)	39.7	39.7	20.6
	Spouse (n = 802)	35.7	48.9	15.5
Education of woman***	None (n = 242)	47.9	43.8	8.3
	Primary (n = 194)	37.1	46.9	16.0
	High school+ (n = 492)	30.1	49.8	20.1
Employment status of woman	Engaged (n = 240)	35.0	44.2	20.8
	Not engaged (n = 688)	36.6	48.8	14.5
Size of household landholdings	50 cents or less (n = 729)	34.3	48.8	16.9
	> 50 cents (n = 199)	43.2	43.2	13.6
Caste of head	SC/ST (n = 363)	38.6	46.6	14.9
	OBC (n = 357)	38.7	46.2	15.1
	Forward (n = 208)	27.9	51.9	20.2

Total	*(n = 928)*	36.2	47.6	16.2

*p < 0.05, **p < 0.01, ***p < 0.001, tested by chi-square.

The results of the models of SHG participation are presented in Table 4. Having no education is associated with a lower odds of being an early or a late joiner, compared to non-participants (reference group). The odds ratio is lower for early joiners than it is for late joiners, suggesting a gradient between education and SHG duration. Having a primary education is associated with a lower odds of being an early joiner compared to non-participants. Women belonging to the middle age category (31 to 44 years) have a higher odds of being an early or late joiner than younger women (under 30 years). These results indicate that education and age are influencing women's self selection into a SHG and confirms the need to control for these characteristics in our analyses.

**Table 4:** SHG participation, by characteristics. Multinomial logistic regression: odds ratios with 95% confidence intervals, using non-member as reference group^a^

Characteristic	Late joiner (< = 2 years) N = 442	Early joiner (> 2 years) N = 150
*Age (ref = 30 years and under) *31–44	2.63 [1.53–4.53]	1.74 [1.18–2.58]
45–59	1.39 [0.73–2.66]	0.92 [0.58–1.44]
*Relationship to head (ref = spouse) *Head	1.52 [0.85–2.72]	0.92 [0.58–1.45]
*Education (ref = high school+) *Primary	0.22 [0.12–0.41]	0.62 [0.42–0.94]
No education	0.57 [0.34–0.98]	0.83 [0.55–1.24]
*Employment (ref = not engaged) *Engaged	1.33 [0.84–2.09]	0.89 [0.63–1.27]
*Landholdings (ref = more than 50 cents) *50 cents or less	1.53 [0.90–2.60]	1.41 [0.97–2.05]
*Caste of head (ref = forward) *OBC	0.69 [0.39–1.19]	0.68 [0.45–1.04]
SC/ST	0.91 [0.52–1.60]	0.80 [0.52–1.24]

^a ^Notes: Results in bold are statistically significant at 5% level, OBC = Other Backward Caste, SC/ST = Scheduled Caste/Scheduled Tribe

### SHG participation and health determinants (A1)

The results of the models of exclusion to health care, exposure to health risks, and male decision-making are shown in Tables 5, 6, 7.

**Table 5:** Models for exclusion to health care. Binomial logistic regression: odds ratios with 95% confidence intervals and goodness of fit statistics^a^

Dependent variable: exclusion to health care (yes, n = 325, no, n = 603)	M0	M1	M2
***Socioeconomic characteristics and caste***			
*Education (ref = high school+) *Primary		0.92 [0.63–1.35]	0.90 [0.61–1.32]
No education		1.40 [0.96–2.05]	1.35 [0.91–1.99]
*Employment (ref = not engaged) *Engaged		0.82 [0.59–1.14]	0.81 [0.58–1.14]
*Landholdings (ref = more than 50 cents) *50 cents or less		**1.52 [1.05–2.20]**	**1.52 [1.05–2.21]**
*Caste of head (ref = forward) *OBC		**1.57 [1.06–2.34]**	**1.52 [1.02–2.27]**
SC/ST		1.08 [0.72–1.63]	1.11 [0.73–1.68]

***SHG participation***			
*SHG (ref = not member) *Early joiner (> 2 years)	**0.56 [0.36–0.86]**		**0.60 [0.39–0.94]**
Late joiner (< = 2 years)	**0.57 [0.41–0.79]**		**0.58 [0.41–0.80]**
(Not member but SHG in household)	**(0.58) ([0.35–0.94])**		**(0.53) ([0.32–0.90])**
Chi square (df) -2 log likelihood	13.3 (3)** 1188.6	24.3(9)** 1177.6	36.4(12)*** 1165.5

Deviation		24.2(3)***	

^a ^Notes: Models are adjusted for age and women's household position. Results in bold are statistically significant, *p < 0.05, **p < 0.01, ***p < 0.001

OBC = Other Backward Caste, SC/ST = Scheduled Caste/Scheduled Tribe.

**Table 6:** Models for exposure to health risks. Binomial logistic regression: odds ratios with 95% confidence intervals and goodness of fit statistics^a^

Dependent variable: exposed to health risk (yes, n = 206, no, n = 722)	M0	M1	M2
***Socioeconomic characteristics and caste***			
*Education (ref = high school+) *Primary		1.24 [0.80–1.93]	1.27 [0.82–1.97]
No education		1.43 [0.92–2.23]	1.50 [0.96–2.35]
*Employment (ref = not engaged) *Engaged		0.96 [0.66–1.39]	0.95 [0.65–1.37]
*Landholdings (ref = more than 50 cents) *50 cents or less		1.46 [0.96–2.21]	1.45 [0.95–2.20]
*Caste of head (ref = forward) *OBC		0.67 [0.43–1.05]	0.67 [0.43–1.05]
SC/ST		0.72 [0.46–1.14]	0.72 [0.45–1.14]

***SHG participation***			
*SHG (ref = not member) *Early joiner (> 2 years)	1.27 [0.81–1.97]		1.35 [0.84–2.18]
Late joiner (< = 2 years)	0.90 [0.64–1.27]		0.99 [0.69–1.42]
Chi square (df) -2 log likelihood	2.3(2) 980.2	51.6(9)*** 930.9	53.6(11)*** 928.9

Deviation		4.0(2)	

^a ^Notes: Models are adjusted for age and women's household position. Results in bold are statistically significant, *p < 0.05, **p < 0.01, ***p < 0.001

OBC = Other Backward Caste, SC/ST = Scheduled Caste/Scheduled Tribe.

**Table 7:** Models for limited decision-making agency. Binomial logistic regression: odds ratios with 95% confidence intervals and goodness of fit statistics^a^

Dependent variable: male decision-making (yes, n = 114, no, n = 814)	M0	M1	M2
***Socioeconomic characteristics and caste***			
*Education (ref = high school+) *Primary		1.23 [0.72–2.11]	1.22 [0.71–2.09]
No education		0.73 [0.39–1.34]	0.72 [0.38–1.33]
*Employment (ref = not engaged) *Engaged		**0.45 [0.25–0.81]**	**0.44 [0.24–0.79]**
*Landholdings (ref = more than 50 cents) *50 cents or less		0.89 [0.53–1.50]	0.90 [0.53–1.52]
*Caste of head (ref = forward) *OBC		1.01 [0.57–1.79]	0.96 [0.54–1.72]
SC/ST		1.35 [0.76–2.40]	1.31 [0.74–2.34]

***SHG participation***			
*SHG (ref = not member) *Early joiner (> 2 years)	1.00 [0.58–1.74]		0.90 [0.53–1.74]
Late joiner (< = 2 years)	0.71 [0.46–1.10]		**0.62 [0.39–0.97]**
Chi square (df) -2 log likelihood	2.8(2) 688.7	51.3(9)*** 640.2	56.5(11)*** 635.0

Deviation		10.4(2)**	

^a ^Notes: Models are adjusted for age and women's household position. Results in bold are statistically significant, *p < 0.05, **p < 0.01, ***p < 0.001

OBC = Other Backward Caste, SC/ST = Scheduled Caste/Scheduled Tribe.

Thirty-five percent of women come from households reporting at least one episode of exclusion to health care. The odds ratios in Model 0 suggest that SHG participation is associated with lower rates of exclusion: compared to the reference group, which in this case is non-participants living in a household without a SHG member, the odds of facing exclusion is significantly lower among early joiners (OR = 0.56, CI = 0.36–0.86), late joiners (OR = 0.57, CI = 0.41–0.79), and non-participants who live in a household with a SHG member (OR = 0.58, CI = 0.35–0.94). Belonging to a household with small landholdings (i.e. less than 50 cents of land) and having OBC affiliations is associated with exclusion (Model 1). Notably, there were no statistically significant differences between forward caste and SC/ST women – who rank the lowest on the caste hierarchy. This finding may be attributed to the poorest tribal group (the Paniyas) not being included in the sample. Model 2 shows that after adjusting for women's characteristics, SHG participation is a significant factor for exclusion to care (deviance = (24.2 (3)). The odds ratios are similar to the estimates of Model 0: the odds of facing exclusion is significantly lower among early joiners (OR = 0.58, CI = 0.41–0.80), late joiners (OR = 0.60, CI = 0.39–0.94), and non-participants who live in a household with a SHG member (OR = 0.53, CI = 0.32–0.90) (M2).

Perceived exposure to health risks was reported by 22% of the women. Exposure to health risks is not significantly associated with any women's characteristic (Model 1) or SHG participation (Model 0 and Model 2).

Globally, there appears to be a high level of decision-making agency, only 12% of women reported male decision-making. As an independent predictor, SHG participation was not significantly associated with decision-making (Model 0). There is a lower odds of reporting male decision-making if a woman is engaged in paid employment (Model 1). Models One and Two are significantly different (deviance = 10.4(2)). After adjusting for women's characteristics, we found a lower odds of reporting male decision-making if women are late joiners (OR = 0.62, CI = 0.39–0.97), but contrary to our expectations, we found no significant associations between decision-making and being an early joiner (Model Two).

### SHG participation and health achievements (A2)

This section presents the results of the binomial logistic regressions for A2 for each of the four health achievements (self perceived health, limitations in ADLs, emotional stress, life satisfaction).

Results for models of self assessed health are presented in Tables 8 and 9. Thirty-five percent of women reported bad health. We found no associations between self perceived health and SHG participation (Model 0). There are significantly greater odds of reporting bad health if a woman is not engaged in paid employment and comes from a household with small landholdings (Model 1). Even after adjusting for women's characteristics, SHG participation is not associated with perceived health (Model 2). As a block, the determinants of health significantly contribute to the model (deviance = 366.0(3)). There are robust associations between bad health and all three health determinants (Model Three). A woman has a significantly greater odds of reporting bad health if she faces exclusion to health care, is exposed to health risks, and reported male decision-making. Limitations in ADLs were reported by 41% of women. The logistic regression results for limitations in ADLs are comparable to those for bad health.

**Table 8:** Models for self perceived health. Binomial logistic regression: odds ratios with 95% confidence intervals and goodness of fit statistics^a^

Dependent variable: Bad health (yes, n = 321, no, n = 607)	M0	M1	M2	M3
***Socioeconomic characteristics and caste***				
*Education (ref = high school+) *Primary		1.18 [0.79–1.76]	1.20 [0.80–1.79]	1.11 [0.70–1.75]
No education		1.49 [1.00–2.23]	**1.53 [1.02–2.31]**	1.37 [0.87–2.17]
*Employment (ref = not engaged) *Engaged		**0.62 [0.43–0.88]**	**0.61 [0.43–0.87]**	**0.61 [0.40–0.91]**
*Landholdings (ref = more than 50 cents) *50 cents or less		**1.55 [1.06–2.27]**	**1.55 [1.06–2.26]**	1.39 [0.91–2.13]
*Caste of head (ref = forward) *OBC		1.00 [0.66–1.52]	1.00 [0.66–1.52]	1.15 [0.71–1.85]
SC/ST		0.93 [0.61–1.43	0.93 [0.60–1.43]	1.04 [0.64–1.70]

***SHG participation***				
*SHG (ref = not member) *Early joiner (> 2 years)	0.97 [0.65–1.44]		1.22 [0.78–1.90]	1.17 [0.70–1.94]
Late joiner (< = 2 years)	0.77 [0.57–1.04]		0.93 [0.67–1.29]	1.02 [0.70–1.47]

***Health determinants***				
*Exclusion (ref = no exclusion) *Exclusion				**1.94 [1.37–2.74]**
*Health risks (ref = no risks) *At least one risk				**10.3 [6.87–15.3]**
*Decision-making (ref = female/joint) *Male				**2.55 [1.57–4.16]**
Chi square (df) -2 log likelihood	3.21(2) 1193.7	143.0(9)*** 1053.9	144.6(11)*** 1052.3	327.6(14)*** 869.3

Deviation		3.2(2)		366.0(3)***

^a ^Notes: Models are adjusted for age and women's household position. Results in bold are statistically significant, *p < 0.05, **p < 0.01, ***p < 0.001,

OBC = Other Backward Caste, SC/ST = Scheduled Caste/Scheduled Tribe.

**Table 9:** Models for Limits in Activities in Daily Living (ADL). Binomial logistic regression: odds ratios with 95% confidence intervals and goodness of fit statistics^a^

Dependent variable: Limits in ADL (yes, n = 377, no, n = 551)	M0	M1	M2	M3
***Socioeconomic characteristics and caste***				
*Education (ref = high school+) *Primary		1.31 [0.89–1.92]	1.31 [0.89–1.92]	1.26 [0.83–1.91]
No education		**1.83 [1.24–2.70]**	**1.82 [1.23–2.71]**	**1.71 [1.12–2.62]**
*Employment (ref = not engaged) *Engaged		**0.64 [0.46–0.91]**	**0.65 [0.46–0.91]**	**0.68 [0.47–0.98]**
*Landholdings (ref = more than 50 cents) *50 cents or less		1.38 [0.96–1.98]	1.37 [0.95–1.98]	1.22 [0.82–1.80]
*Caste of head (ref = forward) *OBC		0.80 [0.54–1.19]	0.80 [0.54–1.19]	0.81 [0.53–1.25]
SC/ST		0.71 [0.47–1.06]	0.71 [0.47–1.07]	0.72 [0.46–1.12]

***SHG participation***				
*SHG (ref = not member) *Early joiner (> 2 years)	0.79 [0.53–1.18]		0.97 [0.62–1.50]	0.91 [0.56–1.46]
Late joiner (< = 2 years)	0.94 [0.70–1.25]		1.14 [0.83–1.57]	1.29 [0.91–1.81]

***Health determinants***				
*Exclusion (ref = no exclusion) *Exclusion				**2.02 [1.47–2.78]**
*Health risks (ref = no risks) *At least one risk				**5.60 [3.84–8.17]**
*Decision-making (ref = female/joint) *Male				**2.23 [1.41–3.52]**
Chi square (df) -2 log likelihood	1.36 (2) 1252.3	129.5(9)*** 1124.1	130.5(11)*** 1123.1	252.5(14)*** 1001.1

Deviation		2.0(2)		244.0(3)***

^a ^Notes: Models are adjusted for age and women's household position. Results in bold are statistically significant, *p < 0.05, **p < 0.01, ***p < 0.001,

OBC = Other Backward Caste, SC/ST = Scheduled Caste/Scheduled Tribe.

Table 10 and 11 shows the results for the markers of mental health. Eighty-eight percent of women reported emotional stress. SHG participation as a sole independent variable was not found to be associated with emotional stress (Model 0). There is a significantly greater odds of reporting emotional stress if a woman is engaged in paid employment and if she comes from a household with small landholdings (Model One). After controlling for women's characteristics, SHG participation significantly improves the model for emotional stress (deviance = 18.6(2)). Inspection of the odds ratios highlight a striking result: although we find no associations between emotional stress and being a late joiner, the odds of reporting emotional stress is significantly lower for early joiners compared to non-participants (OR = 0.52, CI = 0.30–0.93) (Model Two). The determinants of health significantly improve the model (deviance = 55.8 (3)).

**Table 10:** Models for disturbances in mental peace. Binomial logistic regression: odds ratios with 95% confidence intervals and goodness of fit statistics^a^

Dependent variable: disturbances in mental peace (yes, n = 820, no, n = 108)	M0	M1	M2	M3
***Socioeconomic characteristics and caste***				
*Education (ref = high school+) *Primary		1.07 [0.58–1.96]	1.02 [0.56–1.88]	1.07 [0.57–1.98]
No education		0.86 [0.48–1.52]	0.76 [0.42–1.38]	0.71 [0.39–1.31]
*Employment (ref = not engaged) *Engaged		**2.04 [1.13–3.67]**	**2.17 [1.20–3.92]**	**2.29 [1.21–4.04]**
*Landholdings (ref = more than 50 cents) *50 cents or less		**1.90 [1.16–3.12]**	**1.95 [1.18–3.20]**	**1.80 [1.08–3.00]**
*Caste of head (ref = forward) *OBC		1.20 [0.66–2.20]	1.20 [0.65–2.21]	1.14 [0.62–2.12]
SC/ST		0.85 [0.48–1.52]	0.85 [0.47–1.52]	0.86 [0.47–1.56]

***SHG participation***				
*SHG (ref = not member) *Early joiner (> 2 years)	0.64 [0.38–1.10]		**0.52 [0.30–0.93]**	**0.52 [0.29–0.94]**
Late joiner (< = 2 years)	1.29 [0.81–2.04]		1.26 [0.78–2.02]	1.34 [0.82–2.18]

***Health determinants***				
*Exclusion (ref = no exclusion) *Exclusion				**2.98 [1.73–5.13]**
*Health risks (ref = no risks) *At least one risk				1.56 [0.86–2.85]
*Decision-making (ref = female/joint) *Male				**0.52 [0.31–0.89]**
Chi square (df) -2 log likelihood	6.29(2)* 661.2	27.7(9)*** 639.8	37.0(11)*** 630.5	64.9(14)*** 602.6

Deviation		18.6(2)***		55.8(3)***

^a ^Notes: Models are adjusted for age and women's household position. Results in bold are statistically significant, *p < 0.05, **p < 0.01, ***p < 0.001,

OBC = Other Backward Caste, SC/ST = Scheduled Caste/Scheduled Tribe

**Table 11:** Models for life satisfaction. Binomial logistic regression: odds ratios with 95% confidence intervals and goodness of fit statistics^a^

Dependent variable: Unsatisfied in life (yes, n = 99, no, n = 829)	M0	M1	M2	M3
***Socioeconomic characteristics and caste***				
*Education (ref = high school+) *Primary		1.18 [0.66–2.11]	1.10 [0.61–1.97]	1.10 [0.61–1.97]
No education		1.53 [0.85–2.72]	1.32 [0.73–2.38]	1.30 [0.72–2.36]
*Employment (ref = not engaged) *Engaged		1.47 [0.92–2.35]	1.51 [0.94–2.43]	1.57 [0.97–2.54]
*Landholdings (ref = more than 50 cents) *50 cents or less		0.85 [0.50–1.44]	0.89 [0.52–1.52]	0.86 [0.50–1.48]
*Caste of head (ref = forward) *OBC		1.09 [0.60–2.00]	1.06 [0.58–1.94]	1.04 [0.56–1.91]
SC/ST		0.77 [0.40–1.45]	0.76 [0.40–1.45]	0.75 [0.39–1.43]

***SHG participation***				
*SHG (ref = not member) *Early joiner (> 2 years)	**0.34 [0.16–0.73]**		**0.32 [0.14–0.71]**	**0.32 [0.14–0.72]**
Late joiner (< = 2 years)	0.65 [0.42–1.00]		0.68 [0.43–1.08]	0.71 [0.45–1.12]

***Health determinants***				
*Exclusion (ref = no exclusion) *Exclusion				1.23 [0.79–1.93]
*Health risks (ref = no risks) *At least one risk				1.13 [0.69–1.87]
*Decision-making (ref = female/joint) *Male				1.37 [0.70–2.68]
Chi square (df) -2 log likelihood	10.0 (2)** 620.1	22.4(9)** 607.7	32.1(11)*** 598.0	34.0(14)** 596.1

Deviation		19.4(2)***		3.8(3)

^a ^Notes: Models are adjusted for age and women's household position. Results in bold are statistically significant, *p < 0.05, **p < 0.01, ***p < 0.001,

OBC = Other Backward Caste, SC/ST = Scheduled Caste/Scheduled Tribe

Emotional stress is positively associated with exclusion to health (Model 3). The odds of reporting emotional stress is lower if women reported male decision-making (Model 3). After entering the health determinants in the model, the odds ratios for SHG participation remained constant. This indicates that exclusion to health care and decision-making agency are not mediators between SHG participation and emotional stress, but that other explanatory factors, not included in our models, link participation and emotional stress.

Eleven percent of women reported being unsatisfied in life. Interestingly, the odds of being unsatisfied is significantly lower for early joiners (OR = 0.34, CI = 0.16–0.73), but not for late joiners (Model 0). There are no statistically significant associations between life satisfaction and women's characteristics (Model 1). Adding SHG participation to the model after controlling for women's characteristics, significantly improves the model (Model 2) and we find a similar pattern of odds ratios found in Model 0, early joiners are less likely to report being unsatisfied than non-members (OR = 0.32, CI = 0.14–0.71). There are no associations between any of the health determinants and life satisfaction (Model 3).

## Discussion

### Limitations of the study

This study has five main limitations that warrant discussion. The first limitation is the cross-sectional design, which heightened the potential of selection bias. Women self select themselves into a microcredit program, thereby posing a threat to internal validity [5]. Women who have decided to join may exhibit certain characteristics or have preferences that also affect health, leading to spurious outcomes. As in other similar studies, we cannot guarantee that we were able to adequately address selection bias, but we tried to minimize this bias in several ways. First, we modelled the relationship between women's characteristics and their participation, then we controlled for these characteristics through multivariate analysis. Second, we restricted our sample to women who came from households below the poverty line, yielding a more homogenous population of women with respect to education levels, employment status, and living standards. These women were also more likely to have similar reasons for deciding whether or not to join a SHG compared to women who are better off. Third, we asked non-participating households why there was no SHG member in the households and sensitivity analysis confirmed that ill health did not affect our main findings. Fourth, our variable for SHG participation allowed for multiple comparisons. Although, we did not find a gradient in health, we did find that for emotional stress, there was a diverging pattern between early and later joiners, justifying our approach.

The second limitation was that we conducted the study in one Panchayat, selecting non-members living in the Panchayat as the controls; therefore, we cannot exclude the possibility of contamination – changes in norms or practices among female members may spill-over to non-members. For example, several SHGs reported that they had used their savings to help out other women in the community who faced health care costs they were unable to pay for. The dilution of the effects of SHG participation reduces our ability to detect differences between SHG members and non-members, but because SHGs exist across Panchayats and urban areas it was not possible to select controls from an area without a SHG program.

The third study limitation is that we relied on measures of self reported health, which are vulnerable to perception bias [18]. Self assessed health status should not, however, be disregarded. Health is a multidimensional construct that can be viewed through multiple lenses. Self-assessed measures address one limited, but relevant, dimension of health. These measures focus on a person's ability to walk, earn a living, or engage in some social activity, instead of on the underlying pathology of a particular disease [21]. It is important, however to identify key social factors when measuring self-assessed health, such as income, education, access to public health facilities, and perceived social stigma [18,21]. Our prior analysis determined that a deprived and culturally distinct group of tribal women (the Paniyas) were systematically underreporting their health status [9]; therefore, this group was not included in the study. While this reduced the potential for bias in the study, it also precluded an analysis of the group with the greatest need and the most potential to benefit from a development intervention. However, as it is common among microcredit interventions in which the poorest of the poor do not participate, the Paniyas have low participation rates in SHGs. We would; therefore, have had insufficient power to assess the health benefits of their participation.

The fourth limitation is related to our measures of markers of mental health. Our indicators are each based on a single question, which likely did not capture the full breadth of women's emotional stress or life satisfaction. Moreover, these questions have not been validated in the Kerala context, nor is there any evidence that these questions were validated in other studies, which is important in cross-cultural mental health epidemiology [22]. Therefore, our findings need to be interpreted with caution.

The fifth limitation is that we included only women who were either heads or spouses of the heads of households. These women are likely to have more senior roles in the household and greater levels of autonomy compared to other women, which has implications for our findings. For example, lower levels of autonomy among younger women who have never married or who live with their in-laws may, on the one hand, be less likely to join a microcredit program if they are not permitted to join, but on the other hand, may benefit more by participating in a SHG, where they have the opportunity to enhance their financial autonomy and decision-making powers. It is with these limitations in mind that we now discuss the key findings of the study.

### Key findings

The primary finding of this study is that SHG participation appears to offer protection against exclusion to health care; regardless of whether a woman is an early joiner or a late joiner. Moreover, even a woman who does not participate, but lives in the same household as a SHG member faces less exclusion. This is likely attributed to the ability of SHG members, and their household members, to acquire loans to cover health costs when in need – suggested by the high number of loans taken for health purposes. These findings indicate that it is not primarily through an increase in income that exclusion to health care is reduced, as might be expected since SHGs are first and foremost an income-generation strategy. Instead, SHGs are unofficially operating as a coping strategy, helping women to overcome financial barriers and budgetary constraints. Microcredit can be considered as an effective risk mitigation strategy that prevent women from being excluded to health care or falling into debt or impoverishment due to the financial burden of health care. This striking result is congruent with findings of a study conducted in Indonesia; households with better access to financial institutions were better able to smooth their consumption against health shocks [23]. Because microcredit was not designed as a mechanism to protect against exclusion to health care, this may be viewed as an unintended benefit of microcredit participation. In the context of Kerala, where there is a considerable burden of health care costs, this offers an important source of social protection.

Before discussing the policy implications, we outline some other findings of this study, beginning with decision-making agency. We expected that the longer a female participated in a SHG, the less likely she would be to engage in male decision-making, a pattern found in Kerala's neighbouring state of Tamil Nadu [24]. However, male decision-making was reported less among late joiners, but no significant differences were found between early joiners and non-participants. Decision-making agency is difficult to measure and there is no standard approach [24]. Our variable may have inadequately captured the complexities of decision-making agency.

Less exposure to health risks was expected among SHG participants, but no significant differences in exposure to health risks were found between early joiners, late joiners, and non-participants. Although SHG participation may provide opportunities for greater income and informational support that can help to reduce a woman's exposure to health risks [16], this may be insufficient without specific health awareness and education programs (e.g. proper handling of pesticides). Moreover, SHG participants engaging in income-generation activities may face new health risks. It is also possible that our measure of exposure to health risks was not sensitive to women's specific health risks, precluding an adequate assessment of the relationship between SHG participation and exposure to health risks. We know little about the specific health risks that women are exposed to in the Panchayat; therefore, there is a need to better document these risks and their distribution in the population and to devise appropriate interventions.

After controlling for women's characteristics, SHG participation was not found to display any discernable relationship with perceived health or limitations in ADLs. This may be due to a time lag between the intervention and changes in self assessed health [16], although we expected to see improvements among the early joiners. Our results may also be an indication that SHG participation may insufficiently expand women's health opportunities to achieve better self perceived health or improve functionings in their daily activities. Complementary programs or services that address a wider set of women's health determinants may be required, such as awareness campaigns for specific health risks.

Our investigations with respect to markers of mental health paint a different picture. First, among this sample of women, we found a very high rate of women reporting emotional stress, close to 90%, which should be interpreted in light of recent concerns of mental health problems among women in Kerala [14]. Emotional stress was found to be especially concentrated among the poorest women, and more importantly, SHG participation was found to be associated with a lower likelihood of reporting emotional stress and poor life satisfaction. However, another study conducted in Bangladesh, did not find a relationship between women's microcredit participation and their emotional stress [19].

An interesting result from our study is that being an early joiner (but not a late joiner) was associated with lower rates of emotional stress. Women who joined earlier have a greater propensity to obtain different kinds of benefits, such as taking up leadership positions. Elsewhere, it was demonstrated that the prevalence of emotional stress among members declined with increasing duration of membership [19] suggesting that there may be an adjustment period for women after joining a scheme. Microcredit often requires the need for women to engage in new types of activities. "By breaking the barriers of traditional norms and behaviours ascribed to women by patriarchal society, micro-credit may generate anxiety and tension among its recipients" [19, p. 1964]. We cannot, however, exclude the possibility that there may be unobserved heterogeneity between early and late joiners that were unaccounted for in our models. Our study also does not illuminate the specific mechanisms involved in reducing emotional stress – this requires further investigation.

### Policy implications

In addition to the potential unintended health benefits of participating in a SHG, microcredit could serve as a springboard to address local health challenges with complementary or parallel programs [25]. Deciding if and how to integrate microcredit with other health programs or services depends on the local context and health needs of the population. Our study illuminates two possible avenues for building health programs onto the existing SHG networks in Kerala.

First, rising health care costs and inadequate access for the poor suggest a need for social protection mechanisms. We found a large number of loans were reported to be used for health consumption, although loans are supposed to be used for productive activities. The reliance on consumption loans by microcredit participants has been previously noted; various suggestions have been proposed to modify lending practices and incentive arrangements for borrowers [26,27]. Given the robust relationships between SHG participation and exclusion to health care, local government and NGOs may wish to broaden their SHG program to specifically address the health care burden and reduce consumption loans among SHGs. This could be done by "piggybacking" a community health solidarity scheme onto the existing SHG program. Implementing health insurance schemes based on existing community-based or indigenous arrangements have been proposed as one approach to developing community based health insurance [28]. Our action research project currently underway in the Panchayat witnessed a similar demand from the community. SHGs have recently undertaken the development and implementation of a health insurance program. Efforts to launch similar initiatives could benefit other communities in Kerala and warrant further investigation. Such arrangements need to be attentive to design and implementation in order to ensure that the intervention is equitable and effective [29]. In particular, to ensure that women are not overly burdened by the additional commitments of such an insurance scheme, women should be at the forefront in managing and controlling the scheme in order that women's interests are best served.

Second, SHG programs already seem to address some determinants of women's mental health via the provision of social support, protection against financial burdens of health care, and opportunities for income generation. Microcredit could serve as a platform for disseminating education and awareness programs on mental health issues.

## Conclusion

The connections between microcredit and health support the current trend of aligning poverty and health in development policy [2]. Microcredit is increasingly advocated in the global fight against poverty and the second phase of the Microcredit Summit Campaign, which aims to ensure that 100 million of the poorest have access to microcredit, is underway [30]. SHGs have, thus far, received relatively little attention, but these groups have been gathering force across Kerala and other states. SHGs are not a panacea for development [7], but could contribute to improving the health of poor women. The type and extent of health benefits are closely intertwined with the context, type of program and the implementation of the program. In Kerala, women tend to have their basic health needs met, but remain vulnerable to other dimensions of ill health due to the persistence of poverty, financial distress, and gender discrimination. Participation in SHGs can help to ensure that poor women are able to adequately access health care without falling into debt or further impoverishment, while promoting their mental health.

## Competing interests

The author(s) declare that they have no competing interests.

## Authors' contributions

This article is based on KM's PhD dissertation. She conceptualized the paper, analyzed the data, and drafted the paper. SH and DN are the principle investigators on the project in which KM's work is affiliated with. SH was KM's PhD supervisor, he guided her in the data analysis and provided substantive comments on the text. DN facilitated KM's field research and provided substantive comments on the text. All authors read and approved the final manuscript.
